# Water-Repellent Galvanized Steel Surfaces Obtained by Sintering of Zinc Nanopowder

**DOI:** 10.1021/acs.langmuir.3c00182

**Published:** 2023-04-05

**Authors:** Francisco Javier Montes Ruiz-Cabello, Schon Fusco, Pablo Ibáñez-Ibáñez, Guillermo Guerrero-Vacas, Miguel Ángel Cabrerizo-Vílchez, Miguel Ángel Rodríguez-Valverde

**Affiliations:** † Laboratory of Surface and Interface Physics, Department of Applied Physics, University of Granada, Campus de Fuentenueva, Granada ES-18071, Spain; ‡ Department of Mechanics, University of Cordoba, Rabanales Campus, Leonardo da Vinci Building, Madrid-Cádiz Road, km 396, Cordoba ES-14071, Spain

## Abstract

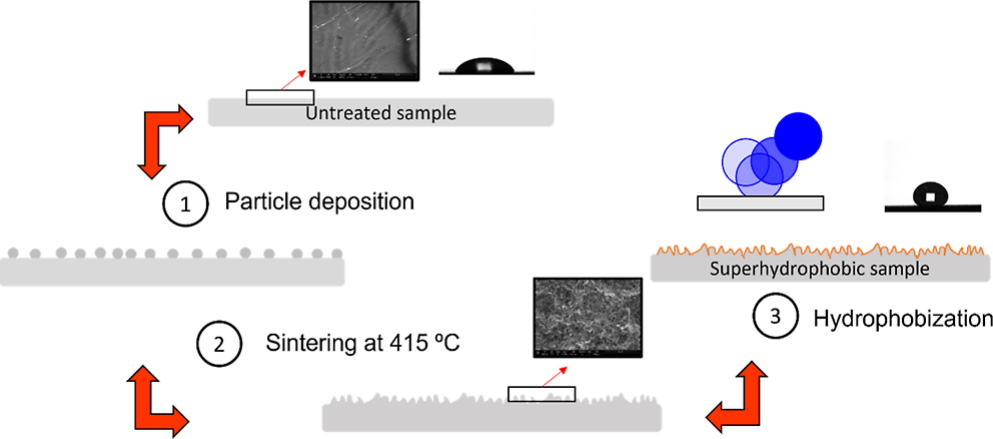

Galvanized steel surfaces are widely used in industry as a solution to prevent corrosion of steel tools that operate in outdoor or corrosive and oxidative environments. These objects are coated with a zinc protective layer deposited by hot dip galvanization. Turning the surface of galvanized steel tools into superhydrophobic may lead to very useful functionalities, although it may be a difficult task, because the preservation of the thin zinc layer is a claim. We propose herein the use of a bottom-up approach based on sandblasting, followed by sintering of zinc nanoparticles on the galvanized steel substrate, which allowed us to produce a zinc-made hierarchical structure required for superhydrophobicity. These samples acquired a double-scale structure that led to superhydrophobicity when they were later hydrophobized with a thin fluoropolymer layer. We found that sandblasting might be useful but not mandatory, unlike the sintering process, which was essential to reach superhydrophobicity. We found that, under certain experimental conditions, the surfaces showed outstanding water-repellent properties. We observed that the sandblasting on galvanized steel caused more damage than the sintering process. Sintering of low-melting-point metal nanoparticles was revealed as a promising strategy to fabricate functional metallic surfaces.

## 1 Introduction

Turning metal-based surfaces into nonwetting surfaces is challenging because metals are high-surface-energy materials. There are many applications for low-adhesion metal surfaces. Lotus-like or superhydrophobic (SH) surfaces have been often proposed as anti-icing,^1,2^ antibiofouling,^3^ antibacterial,^4^ or noncorrosive solutions.^3,5−7^ Water-repellent surfaces are obtained when specific surface texture and low-surface energy compounds are incorporated on the surface. However, most manufactured metallic surfaces are smooth and hydrophilic. Producing SH surfaces on those materials requires the use of bottom-up approaches, such as surface coatings.^8−10^ Unfortunately, most of the commercially available SH coatings are not durable^11^ or may mask other interesting surface functionalities of the material. For this reason, an alternative strategy is the use of top-down approaches. They are based on a surface modification aimed to incorporate topographic features, followed by an increase of the intrinsic contact angle through the deposition of a thin low-surface energy layer.^12,13^ The physical and chemical modification of the metal surface may be achieved by one-step or two-step strategies.

Galvanized steel (GS) surfaces are zinc-coated steel/iron surfaces, typically fabricated by hot-dip galvanization. Depending on the galvanization process, the thickness of the zinc coating may vary from microns to millimeters.^14^ The main purpose of the incorporation of the zinc coating on steel is the corrosion or oxidation prevention of the bulk steel. This process is less expensive than the fabrication of stainless steel. For this reason, GS is used in many industrial applications that require large steel tools:^14^ building structures, roofs, gratings, sheets, and wires. These elements operate in wet/humid conditions, and the incorporation of nonwetting properties might be particularly beneficial.

However, GS is a coated material, and the preservation of the zinc coating is a must. This is an issue if the SH surfaces are prepared by using top-down approaches because these strategies require the material removal intended to create a specific surface texture.

Some of us proposed a protocol to fabricate water-repellent GS following a top-down route based on sandblasting and soft acid etching.^12^ New bottom-up approaches have been reported to produce SH surfaces on GS.^1,8^ The incorporation of surface composites is still one of the most recurrent strategies.^10^ Other options are based on the incorporation of enhanced zinc-based coatings on steel. These coatings are created by electrodeposition followed by chemical modification of steel surfaces.^1^ However, there is still a gap in the state-of-the-art approaches. Surface treatments that directly incorporate the SH properties on GS are challenging.

In this work, we propose a scalable bottom-up strategy to fabricate SH surfaces on GS. The strategy is based on a combination of two different texturization methods: sandblasting and nanoparticle (NP) sintering, both aimed to create the hierarchical surface structure on GS. With these two roughening methods, a double-scale surface texture is produced with minor damage to the zinc layer. Sintering of micro-/nanoparticles has been used in other studies to fabricate SH surfaces.^15^ Ling et al.^16^ fabricated transparent SH films by sintering silica NP onto glass surfaces. Their route to create the nanoscale structure was divided into two steps: a deposition process in which silica NPs are spontaneously adsorbed on the surface by electrostatic interactions and a second process in which the particle–substrate bonding is enhanced with a sintering process at temperatures close to the melting point of silica. Our study is inspired by that work. We found that the incorporation of zinc NP on the GS surface led to superhydrophobicity, once it was further coated with a thin fluoropolymer layer.

We followed two different routes to create direct surface roughness: (a) sandblasting followed by the sintering process of zinc NPs and (b) direct sintering on the GS surface. Sintering was used since, in a previous study,^12^ we found that this roughening method was determining to produce SH samples on GS. The fabricated samples are robust, as revealed by several durability tests conducted on them. These tests are presented in a separate section within the Supporting Information.

## 2 Materials and Methods

### 2.1 Sample Preparation

**Figure 1:**
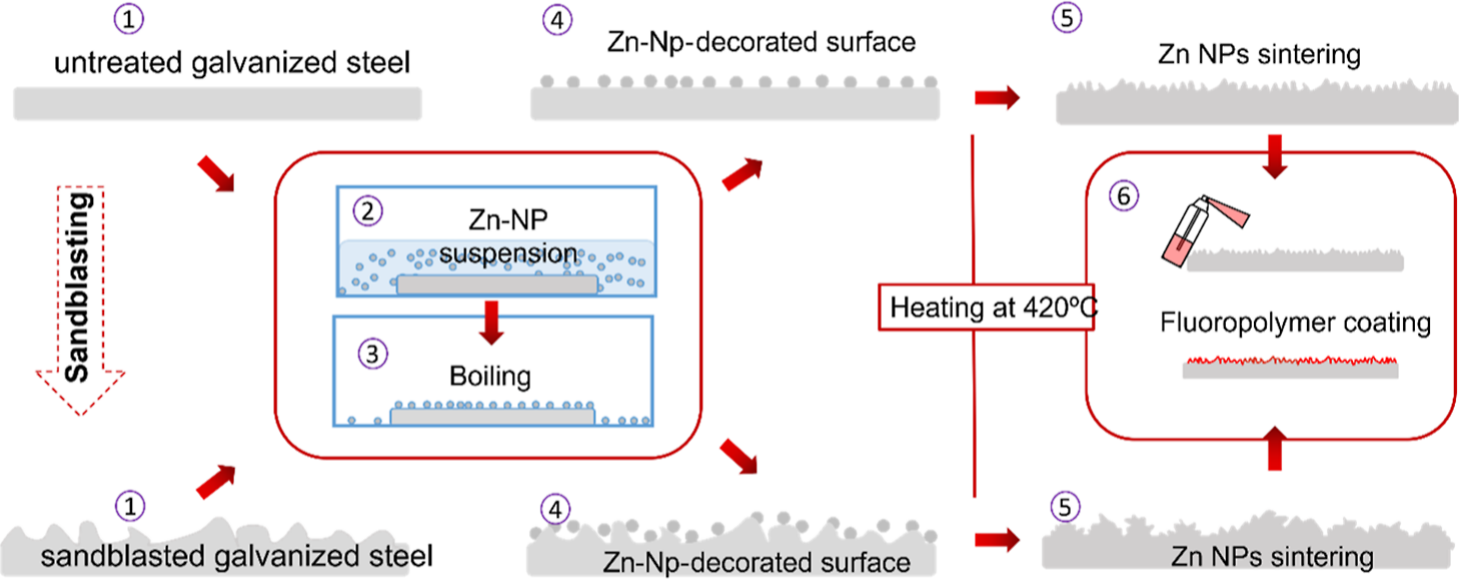
Routes for the fabrication of superhydrophobic galvanized steel surfaces with and without a previous roughening process by sandblasting. In all cases, the sintering process is carried out by heating the sample at 420 °C and further hydrophobization with a Dupont AF solution.

The GS sheets were supplied by Modulor GmbH (Germany) in sheets of 250 × 500 mm^2^ and 0.5 mm in thickness, and then cut into smaller pieces of (25 × 25) mm^2^. As determined in a previous work, the zinc layer covering the steel bulk of the samples is around 16 μm thick.^12^ Before any surface modification, the samples were washed with alkaline detergent, rinsed with abundant distillate water, and dried at room temperature. In Figure 1, we illustrate the treatments followed to prepare the samples. These procedures are divided into two main groups: a group of samples that received a prior sandblasting treatment and another group with no previous sandblasting.

#### 2.1.1 Sandblasting

The sandblasting was carried out in a Sandblast Cabinet-CAT990 (MW-TOOLS) using brown corundum RBT9 100 (MPA, Spain) of grain sizes 106–150 (μm). The exposure time was fixed at 10 s, the pressure was adjusted to 0.3 MP, the separation distance fixed at 20 cm, and inclination angle was set at 90°. Sandblasting allowed us to create micro-sized surface features. We were able to fabricate surfaces with an average roughness that was always greater than Ra = 4 μm. According to our previous work,^12^ this minimum roughness value reveals a balance to ensure superhydrophobicity without significant surface damage induced by sandblasting. The optimal texture was accomplished once other nanoasperities were later incorporated into the surface structure. In this work, the nanodefects were incorporated by the deposition of zinc NPs followed by sintering.

#### 2.1.2 Sintering of Zinc Nanoparticles

This process was carried out on both the sandblasted and untreated GS samples. The samples were air-plasma cleaned in a Plasma Ascher (EMITECH K1050X) operating at 50 W and low pressure (10^–1^ mbar) for 10 min. The plasma treatment allowed us to clean and activate the surface for promoting the liquid spreading. Afterward, the samples were placed inside a glass Petri dish (60 mm diameter). Several colloidal suspensions of zinc nanopowder (average particle size: 40–60 nm, Sigma-Aldrich) were prepared in acetone. The concentration of the zinc nanopowder was adjusted between 0.2 and 10 g/L. The acetone was chosen as a volatile solvent because the suspensions were very stable, and the coffee-ring effect was mitigated.^17^ For each concentration, we prepared 5 mL of solution that was poured into the Petri dish. The Petri dish with the sample fixed on its bottom was introduced inside the oven at 100 °C. In a few seconds, the suspension started to boil, and once the solvent was fully evaporated, a uniform nanopowder film was formed on the sample. The sample was gently removed from the Petri dish and placed in the oven at room temperature. The oven was then turned on until the temperature inside reached the melting point of 415 °C. Once this temperature was reached, the oven continued working for 5 min and then turned off. Finally, the sample was cooled down to room temperature, removed from the oven, and introduced again in the plasma cleaner, using previous settings. The purpose of the plasma treatment was surface activation to improve the later fluoropolymer coating deposition.

#### 2.1.3 Hydrophobization

The hydrophobization was conducted by spraying a mixture of Dupont AF1600 (Chemours) dissolved in a fluorocarbon solvent FC-72 (3 M) in a of ratio 1/20 (v/v). A first layer was applied, and the sample was let dry. Subsequently, it was again sprayed with the same solution, but the sample was now introduced into the oven at 100 °C for 10 min. This curing step is aimed at producing a more robust and durable coating, as recommended by the supplier.

### 2.2 Wetting Analysis

#### 2.2.1 Bouncing Drop Experiments

Bouncing drop experiments allowed us to qualitatively distinguish the degree of water repellency between SH samples.^18^ These experiments are based on the monitoring, by means of a high-speed camera, of a drop that bounces on a repellent surface once it has been released from a fixed height. The number of bounces is ruled by the dissipation induced by the drop-surface tensile adhesion. Hence, the greater the number of bounces, the lower the adhesion. The release height was (10.1 ± 0.2) mm, and the drop volume was typically 4 μL. Under these conditions, the Weber number before the first impact was estimated to be We ≅ 4. The entire sequence was recorded with a high-speed camera (Phantom Miro 4, AMETEK) at 3000 fps.^18^ A full bounce is identified when the drop is completely separated from the surface after each impact.

#### 2.2.2 Tilting Plate Experiments and Contact Angle Measurements

The tilting plate experiments are useful to analyze the shear adhesion of drops on surfaces.^19^ Details on the experimental procedure are given elsewhere.^18^ In our experiments, a 50 μL drop is deposited on the surface, previously fixed to an inclinable platform. Initially, the platform is placed horizontally, and the drop remains static. Under these conditions, the drop shape is expected to be axisymmetric, which means that the contact angle is constant along the entire contact line. Afterward, the platform is inclined at a fixed rate of 5°/s, and the acquisition rate is 5 fps. The inclination process breaks the initial drop symmetry. The observable contact angle depends on the contact line point, with the lowest value observed at the uphill point and the highest one observed at the downhill point.^20^ Depending on the wetting properties of the sample, during the early tilting process, the drop contact line remains static, but when a certain tilt angle is reached, the contact line starts to move partially. In most cases, the downhill point moves first,^21^ followed by the uphill point. This is because the initial drop is generally formed close to the advancing mode.^21^ These experiments may serve to determine the advancing and receding contact angles (RCA) as well as the CTA, which is defined as the minimum tilt angle for which a global motion of the drop is observed.^22^ However, in this work, this technique was only used for the determination of the CTA. The measurements of contact angles on SH surfaces, based on goniometry methods are a very difficult task and may not be useful to distinguish the degree of superhydrophobicity. This happened with the tilting plate experiments. For this reason, the contact angles of the most relevant samples were determined with low-rate dynamic contact angle measurements based on an axisymmetric drop shape analysis profile (ADSA-P).^23^ A microinjector (Hamilton PSD3) is used to vary (from below) the water volume of a drop formed over a drilled sample. The experiment is divided into two different processes: a growing process in which the drop volume increases at a constant flow rate of 2 μL/s until a volume of 200 μL is reached. The second process is a shrinking process in which the drop volume is decreased to 20 μL. The advancing contact angle (ACA) is estimated during the drop growing process from the average of the contact angles observed when the drop advances on the surface. The RCA is calculated by averaging the contact angles observed during the receding of the drop contact line (drop shrinking).

### 2.3 Roughness and Surface Morphology Analysis

The microscale surface roughness was analyzed with white-light confocal microscopy (Plu Sensofar, Spain). We focused on the estimation of the average roughness (Ra) of the samples. This parameter was calculated by averaging the different Ra values measured with, at least, 5 topographies acquired in different locations on each sample. We used a magnification of 50X, the scanned area was (285 × 210) μm^2^, and the z-step was 0.2 μm.

The surface morphology of the samples was also analyzed with high resolution environmental scanning electron microscopy (FEG-ESEM Quanta-650F), operating at 5 kV and high vacuum. We chose two different magnifications of 500X and 4000X aimed to analyze the surface structure at two different scales.

### 2.4 Chemical Analysis

The chemical analysis was conducted by combining standard ESEM imaging with chemical mapping by EDX (FEI QUANTA 650FEG). We focused on the presence of three different elements: Zn, Fe, and Al. This analysis is aimed at determining whether the roughening methods damage the bulk material or affect the surfaces chemical composition. In addition, an XPS analysis was also conducted. This analysis was aimed at determining, in more detail, whether the heating process modified the chemical composition of the GS (the results are shown in the Supporting Information).

## 3 Results and Discussion

### 3.1 Effect of the Heating Treatment

The heating treatment was used to incorporate zinc NPs onto the GS by sintering. However, in preliminary experiments, we observed that this process (without any particle deposition) induced changes in the surface texture of GS. For this reason, we first investigated the role of the heating treatment in the final surface roughness and wettability of the surfaces (once they were hydrophobized). With this analysis, we validated if the heating itself was able to reach the surface texture that is required to produce SH surfaces on GS.

**Figure 2:**
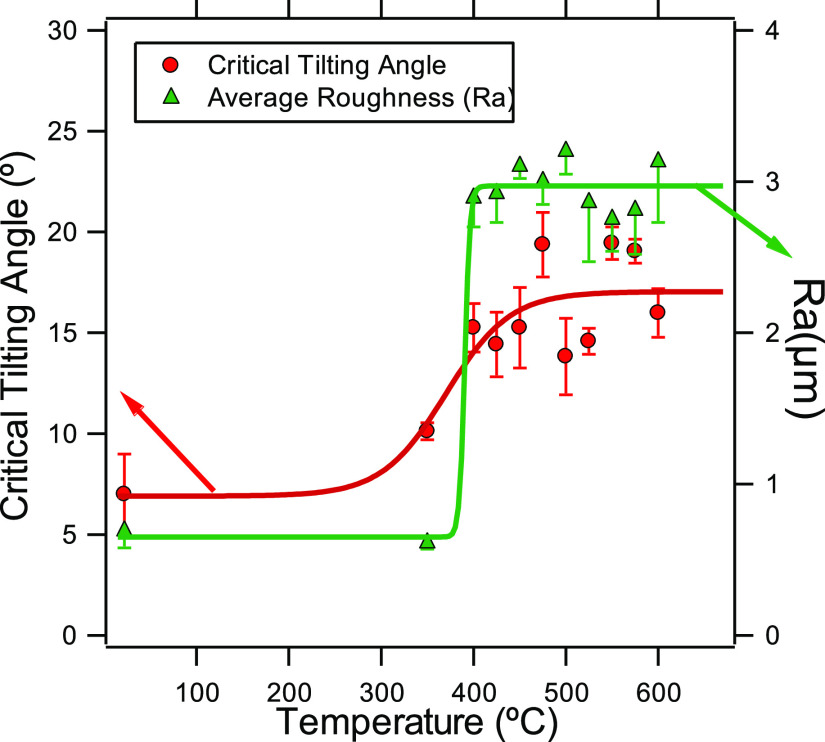
Critical tilting angle and average roughness factor (Ra) in terms of the heating temperature. In this graph, we also included the results for the unheated sample for comparison. Lines are guides to the eye.

We prepared 10 different GS samples by heating for 5 min the previously cleaned as-received samples at temperatures ranging from 350 to 600 °C. Once the samples were cooled down, they were hydrophobized. For comparison, we included in the same graph the results that were obtained for an untreated (not heated) but hydrophobized sample. We focused on the Ra values and compared them with the CTA values obtained for each temperature. Results are plotted in Figure 2.

**Figure 3:**
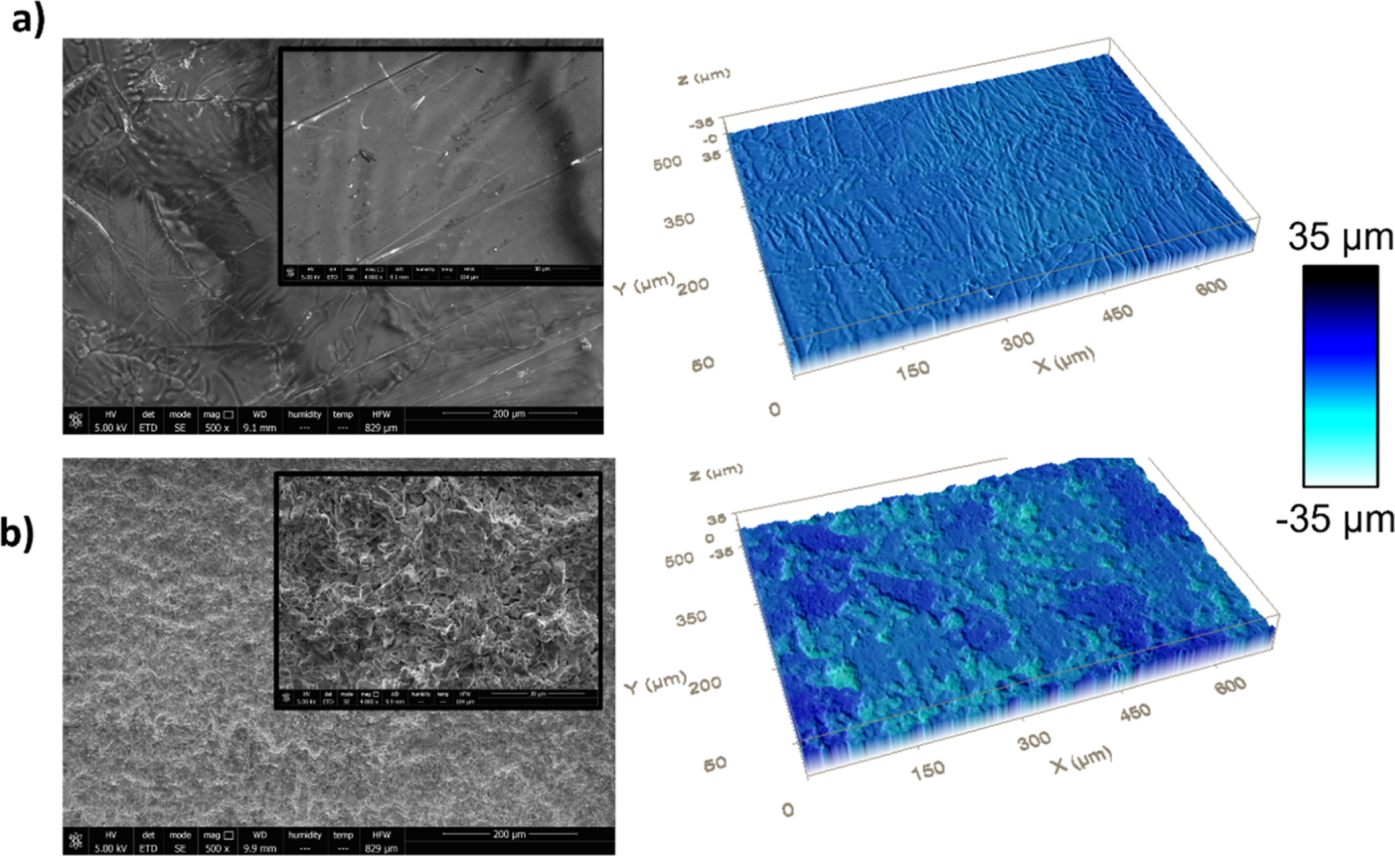
SEM images (left) and confocal topographies (right) obtained for (a) untreated sample and (b) heated sample at 420 °C.

Above a certain temperature value, close to the zinc melting point (around 415 °C), the heating process was able to increase the surface roughness. A more detailed analysis of the surface structure induced by heating will be discussed below. When the heat treatment is conducted at temperatures lower than 400 °C, no significant changes in the surface structure and wettability properties of the samples are observed. However, above 450 °C, we did not notice any clear dependence of temperature on the final roughness. This was also found with the wetting results. Besides, above a certain temperature, the critical tilting angle was higher than the one measured for the smooth (unheated) samples. This reveals that the heating process, even though it increased the surface roughness, was still unable to create the specific surface structure, leading to superhydrophobicity. Similar conclusions related to sandblasting were drawn in a previous work;^12^ in Figure 3, we show images of the surface structure analyzed by ESEM at two different scales. We also plot the surface topographies captured by confocal microscopy. The structure of an untreated sample (Figure 3a) is very different from that of a heated (at 420 °C) surface (Figure 3b). The influence of the thermal treatment on the roughness and surface morphology of the GS samples is noticeable, as discussed above.

A proper sintering process requires the use of sintering temperatures very close to the melting point of the NP material. According to the results of the study reported in this section, we fixed the sintering temperature to 420 °C. Higher temperatures did not influence the final roughness of the samples. As reported in similar studies aimed at producing SH surfaces by sintering NPs,^16^ temperatures higher than the melting point of the material may induce a total melt of the NP, which might reduce the surface roughness at the nanoscale.

### 3.2 Effect of the Sintering Process

**Figure 4:**
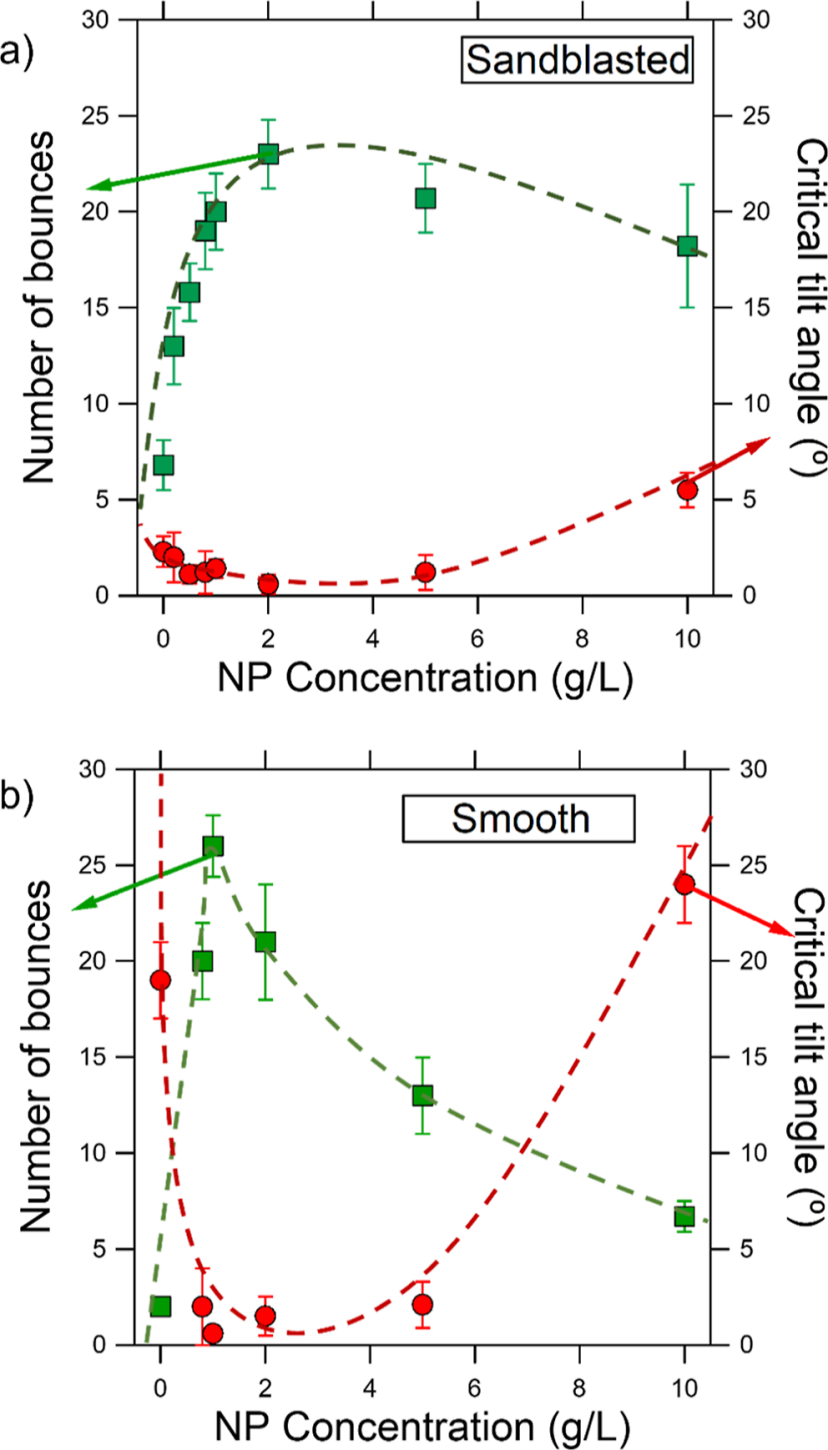
Wetting properties of the modified GS surfaces used in this work, characterized by bouncing drop (squares) and tilting plate experiments (circles). (a) results for the sandblasted + sintered GS samples and (b) smooth + sintered GS samples. The results are shown in terms of the nanoparticle (NP) concentration of the 5 mL solution used to cover the surface prior to heating it. Dash lines are guides to the eye.

In this section, we explored the wettability properties of all the samples produced by sintering Zn NPs. The analysis was conducted for two set of samples: smooth and sandblasted samples. As discussed above, sandblasting and thermal heating, despite being able to change noticeably the surface roughness and wetting properties of the GS samples, were insufficient to generate the specific texture that is required for superhydrophobicity. The samples used in this work were all hydrophobized. This further functionalization is mandatory because the sintered samples were extremely wettable. The goal of the study presented in this section is to find the optimal particle concentration needed to enhance the water repellency for each type of treatment. For this purpose, we used different colloidal suspensions of Zn NPs at concentrations varied up to 10 g/L. We explored the degree of superhydrophobicity using tilting plate experiments and bouncing drop experiments. The use of both techniques was aimed at characterizing both the shear and tensile drop adhesion. In Figure 4a, we show the results for those samples that were previously sandblasted, while in Figure 4b, we show the results for the smooth samples.

Comparing Figure 4a with Figure 4b, we concluded that the sandblasting promotes the water repellency since, for the entire range of NP concentration, the critical tilting angle was always lower than 10°. However, for the smooth samples (no previous sandblasting), the critical tilting angle may reach values higher than 20° for some particle concentrations. In contrast, the best results, in terms of superhydrophobicity for all the prepared samples, were found for the sintered smooth sample using a solution of zinc nanopowder at 1 g/L. This was confirmed by the high number of bounces (26 ± 2) and the low critical tilting angle (0.6 ± 0.3°). The sintered sandblasted sample that showed the best performance was fabricated using a concentration of zinc nanopowder of 2 g/L. In this case, the measured number of bounces was 23 ± 2 and the critical tilting angle was 0.6 ± 0.5°. The differences are not significant, and both samples revealed excellent water repellency properties. To confirm their superhydrophobicity, the wettability of these two selected samples was also analyzed by drop shape analysis (ADSA-P) with growing-shrinking experiments. These experiments provide the contact angle values collected in Table 1.

**Table 1:** ACA, RCA, Critical Tilting Angle, and Number of Bounces Measured for the Samples That Revealed the Best Results in Terms of Water Repellency among All the Samples Analyzed Using Each Procedure

sample	ACA (°)	RCA (°)	critical tilting angle (°)	# bounces
sandblasting + sintering NPs (2 g/L)	158 ± 3	154 ± 2	0.6 ± 0.5	23 ± 2
sintering NPs (1 g/L)	164 ± 2	161 ± 2	0.6 ± 0.3	26 ± 2

We found that the range of optimal nanopowder concentration is shorter for the smooth surfaces than for the sandblasted ones, but sandblasting is not essential to reproduce water-repellent properties.

It is worth mentioning that the surface roughness at the microscale was not much influenced by the particle concentration. In Table 2, we show the Ra values for the most representative samples of this study. In this table, we also included the measured Ra values for nonheated samples as well for comparison. Our first conclusion is that the thermal heating increases the roughness, regardless of the Zn NPs. As illustrated in the previous section, this increase is especially clear for smooth samples since the roughness increased by a factor of 5 after heating the sample. Concerning the role of NP concentration, we observed that for low concentrations, the roughness scales with the NP concentration, but it decreases again at high concentration values. This might be expected since the addition of a roughening agent does not always lead to higher roughness.^18^ Above a certain degree of roughness, a roughening agent may no longer be considered as such. Similar conclusions were drawn in our previous work related to the etching time used to create nanoasperities: at short acid exposure, the roughness increased with etching time, but the opposite trend was observed for longer acid etching.^12,18^

**Table 2:** Average Roughness Values (Ra) for the Most Representative Samples Used in This Work

	sandblasted samples	smooth samples
nanopowder concentration	Ra (μm)	Ra (μm)
0 g/L (not heated)	5.3 ± 0.3	0.69 ± 0.11
0 g/L	8.9 ± 1.3	3.3 ± 0.3
1 g/L	10.2 ± 1.2	3.6 ± 0.4
2 g/L	9.1 ± 1.1	2.9 ± 0.2
5 g/L	4.4 ± 0.4	4.5 ± 0.3

### 3.3 Surface Morphology and Chemical Composition

**Figure 5:**
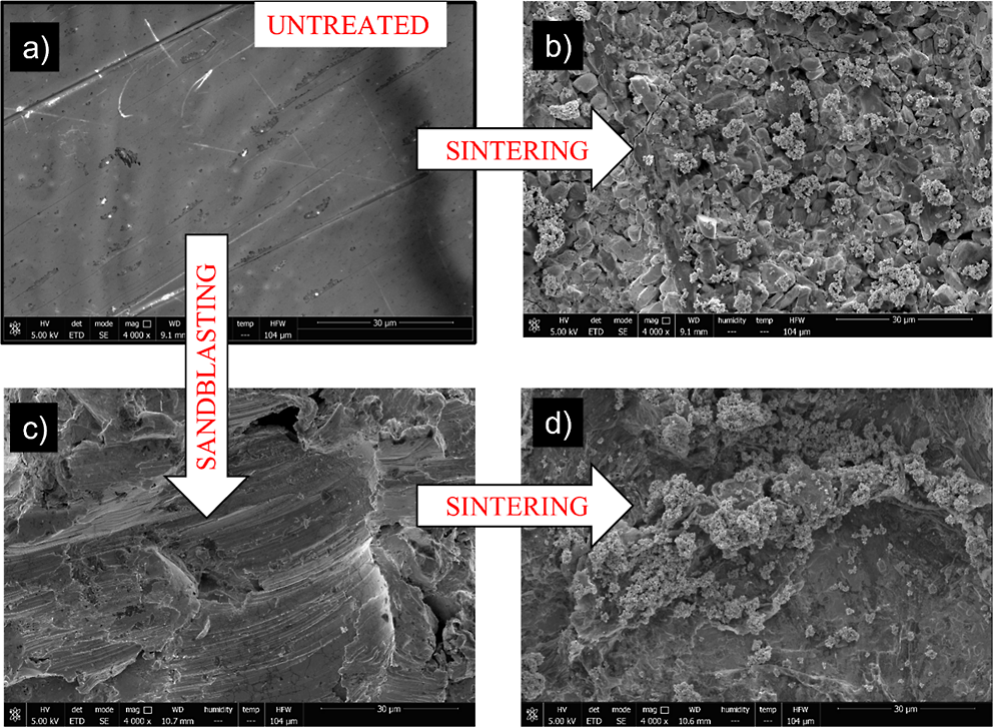
ESEM images of four representative samples: (a) untreated sample, (b) a smooth surface that was subsequently sintered, prior deposition of a 1 g/L solution of zinc NPs, (c) a sandblasted surface, and (d) sandblasted + sintered surface, prior deposition of a 2 g/L solution of zinc NPs. The samples in (b,d) were those that showed the best results in terms of water repellency.

The morphology of the samples was studied by ESEM. The goal is to analyze the micro-/nanostructure incorporated on the GS surfaces after roughening with the most efficient treatments. In Figure 5, the ESEM images of four representative samples are shown: (a) A smooth sample (untreated), (b) a smooth sample further heated to sinter Zn NPs (1 g/L colloidal suspension), (c) a sandblasted sample, and (d) a previously sandblasted sample treated by sintering Zn NPs on it (2 g/L colloidal suspension). The effect of surface heating on GS was analyzed in detail in Section 3.1. In Figure 5, it is clear how the heating and sintering processes incorporate a hierarchical structure based on micro- and nanosized defects. These structures validate the excellent water repellency properties of the samples referred to in Table 1.

**Figure 6:**
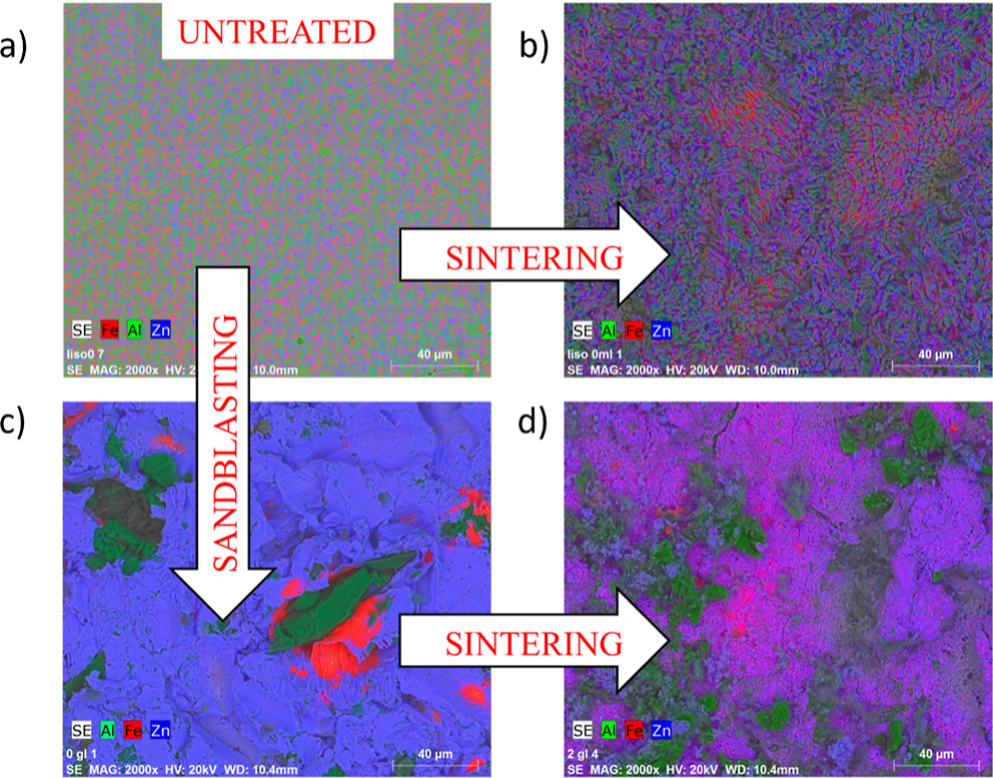
Mapping obtained by ESEM-EDX: (a) an untreated sample, (b) a smooth + sintered sample, (c) a sandblasted sample, and (d) a sandblasted + sintered sample. The green signal corresponds to Al, the red signal to Fe, and the blue signal to Zn.

The next step was to study how each surface roughening method affected the chemical composition. The analysis was conducted through a chemical mapping focused on the presence of three different elements dominant on the GS surfaces: Fe, Al, and Zn. This analysis points out to the damage that each surface treatment caused on the samples. In Figure 6, we show the chemical mappings of the same samples used in Figure 5. The smooth (untreated) sample reveals a very homogenous distribution of the three elements. The lower contrast observed for the untreated sample may be explained in terms of a lower surface roughness since the picture is a superposition of the ESEM image and the chemical mapping. A very similar elemental chemical distribution was observed for the smooth + sintered sample (Figure 6b). Here, the blue signal is more pronounced due to a higher Zn concentration. This is expected due to the presence of sintered nanopowder. Otherwise, the homogeneous chemical distribution was no longer observed for the rest of the samples in Figure 6. The inhomogeneity is particularly relevant to the sandblasted sample (Figure 6c). Some microsized corundum alumina particles embedded in the sample are clearly distinguishable. These particles are surrounded by material rich in iron (confirmed by a red corona around the particle). This is an indication of a possible removal of the native Zn coating caused by the particle impact. Once the sample is subjected to the sintering treatment (Figure 6d), the chemical homogeneity is partially restored. However, we can still observe the presence of alumina particles and a higher presence of iron on the surface if we compare it with the untreated GS (Figure 6a) or the smooth + sintered surface (Figure 6b). These results indicate that sandblasting does not ensure that GS surfaces remain unaltered. Surface texturization by single sintering (without any prior sandblasting) is the most convenient strategy because it is equally effective in terms of wetting response but less harmful than combining it with sandblasting. The chemical composition of the studied samples was further analyzed by XPS, comparing it with an untreated GS surface. The aim is to demonstrate that the structured sample maintains a very similar composition to the original one. In particular, the presence of Fe on both the untreated and smooth + sintered samples is negligible, which suggests that the protective zinc layer remains unaltered in both cases. The results are shown in the Supporting Information (see Supporting Information for more details).

In this work, we showed two different routes to fabricate water-repellent surfaces on GS. Both routes are focused on the incorporation of double-scale roughness and a further hydrophilization by fluoropolymer deposition. The difference between both strategies was the treatment used to create roughness on the surface: the first one consisted of a combination of sandblasting + sintering Zn NPs, while the second one was only based on sintering Zn NPs. Although we observed that the heating during the sintering process modified the surface roughness (higher roughness parameter and greater drop adhesion), the incorporation of the nanopowder was essential. Unlike our previous study with similar purposes,^12^ here, we concluded that sandblasting is unnecessary, although it allows us to find the experimental conditions that ensures water repellency. In contrast, no sintering is needed because both the sandblasting and the thermal heating by themselves, were not able to create the proper surface texture. Besides, we also observed that sandblasting was harmful when the chemical composition of the surface was analyzed in detail. The thermal heating was less aggressive and led to the same degree of water repellency. On the other hand, NP sintering is postulated as a nonaggressive bottom-up approach to fabricating SH surfaces on GS.

## 4 Conclusions

The sandblasting process increases the surface roughness remarkably at the microscale. However, we observed that this texture is not enough to reach extreme water repellency. An additional treatment aimed at adding nanosized surface asperities is also required. This finding agrees with a previous study on GS surfaces as well.^12^ In that work, the nanoasperities were created by a soft acid etching. In this study, we changed it by a less aggressive method based on the sintering of zinc NP.

The NP sintering process modified the surface structure in two ways: the thermal treatment needed to melt the Zn NPs altered the roughness at the microscale. However, we observed that, like sandblasting, the roughening induced by heating was not enough for the second level of roughness incorporated by the Zn NPs that is essential for superhydrophobicity. Sandblasting allowed us to find out the experimental conditions required for superhydrophobicity during the optimization of the sintering process. However, we found that sandblasting damaged the GS surface at the microscale. The treatment based on NP sintering seems to be more convenient because of its excellent water-repellent performance and minor changes in the original chemical composition of GS.

NP sintering to produce water-repellent surfaces has been proposed in other works, specifically those aimed at fabricating durable and superhydrophobic silica surfaces.^16,24^ However, scarce works^24^ use this strategy to create a specific nanostructure on metal surfaces since most metal particles have high melting points. NP sintering is a promising strategy to create hierarchical structures on surfaces with relatively low melting points.

This work proposes the use of sintered metal nanoparticles to incorporate a hierarchical texture on metal surfaces. This strategy was not used earlier and opens up new possibilities for fabricating robust functional surfaces, such as lotus-like or petal like surfaces. We chose a fluoropolymer deposition as the hydrophobization method, although it can be replaced by any other hydrophilization method capable of creating a uniform and thin low-energy coating.^18^ In fact, the use of fluorinated compounds should be avoided due to their toxic nature. In addition, the durability tests confirm that the life of the coating might be increased by using a hydrophobic compound which is more strongly adhered to the substrate. However, the focus of this work is on the texturization method rather than the hydrophobization method.

## Supporting Information Available

Chemical analysis of the samples by XPS and durability tests (PDF)

## Abbreviations

SH: superhydrophobic
ADSA-P: axisymmetric drop shape analysis-profile
ESEM: environmental scanning electron microscopy
GS: galvanized steel
EDX: energy-dispersive X-ray spectroscopy
NP: nanoparticle
ACA: advancing contact angle
RCA: receding contact angle
CTA: critical tilting angle
