# Supplementary material for: Water-Repellent Galvanized Steel Surfaces Obtained by Sintering of Zinc Nanopowder
Source: Langmuir. 2023 Apr 5;39(15):5469–76. doi: 10.1021/acs.langmuir.3c00182 (PMC10116646; doi:10.1021/acs.langmuir.3c00182)
Supplement: Supplementary file 1 — la3c00182_si_001.pdf [file la3c00182_si_001.pdf]

Supporting Information

Water-repellent galvanized steel surfaces obtained by sintering of zinc nanopowder

Francisco Javier Montes Ruiz-Cabello[1], Schon Fusco, Pablo Ibáñez-Ibáñez[1], Guillermo Guerrero-Vacas[2], Miguel Ángel Cabrerizo-Vílchez[1], Miguel Ángel Rodríguez-Valverde[1]*

[1] Laboratory of Surface and Interface Physics, Department of Applied Physics, University of Granada, Campus de Fuentenueva; ES-18071 Granada, Spain.

[2] Department of Mechanics, University of Cordoba, Rabanales Campus, Leonardo da Vinci Building, Madrid-Cádiz Road, km 396, ES-14071 Cordoba, Spain

*Corresponding author:

E-mail: fjmontes@ugr.es

Applied Physics Department, Faculty of Sciences, Campus de Fuentenueva; E-18071 Granada, Spain

Tel: +34-958240771

Fax: +34-95824321

Table of contents

- Chemical analysis of the samples by X-ray Photoelectron Spectroscopy (XPS)

- Durability test of the samples

1. Chemical analysis of the samples by XPS

In this supplementary document, the XPS spectra of 3 representative samples are shown, for comparison. We chose the following samples: a) untreated galvanized steel sample (control), b) a galvanized steel surface that was not previously sandblasted that was roughened by zinc NP sintering (namely, NP sintered) and c) a NP-sintered sample with an amorphous fluoropolymer coating (NP sintered +AF). The sintered samples used in b) and c) were fabricated using a solution of a 1g/L of zinc NP. As discussed in the main document, that roughening method offered the best wetting performance among all the samples fabricated in this study, once the surface was further hydrophobized with the fluoropolymer solution.

The sandblasted samples were not analyzed here, because the EDX analysis on these samples revealed a damage induced by the particles impact. In addition, the wetting results of, either the selected samples previously sandblasted or those that were not sandblasted were comparable, so we concluded the sandblasting might be avoided.

Our XPS analysis was focused on detecting the presence of four relevant chemical elements: Zn-2p, Fe-2p, O-1s and F-1s. Results are displayed in Figure S1

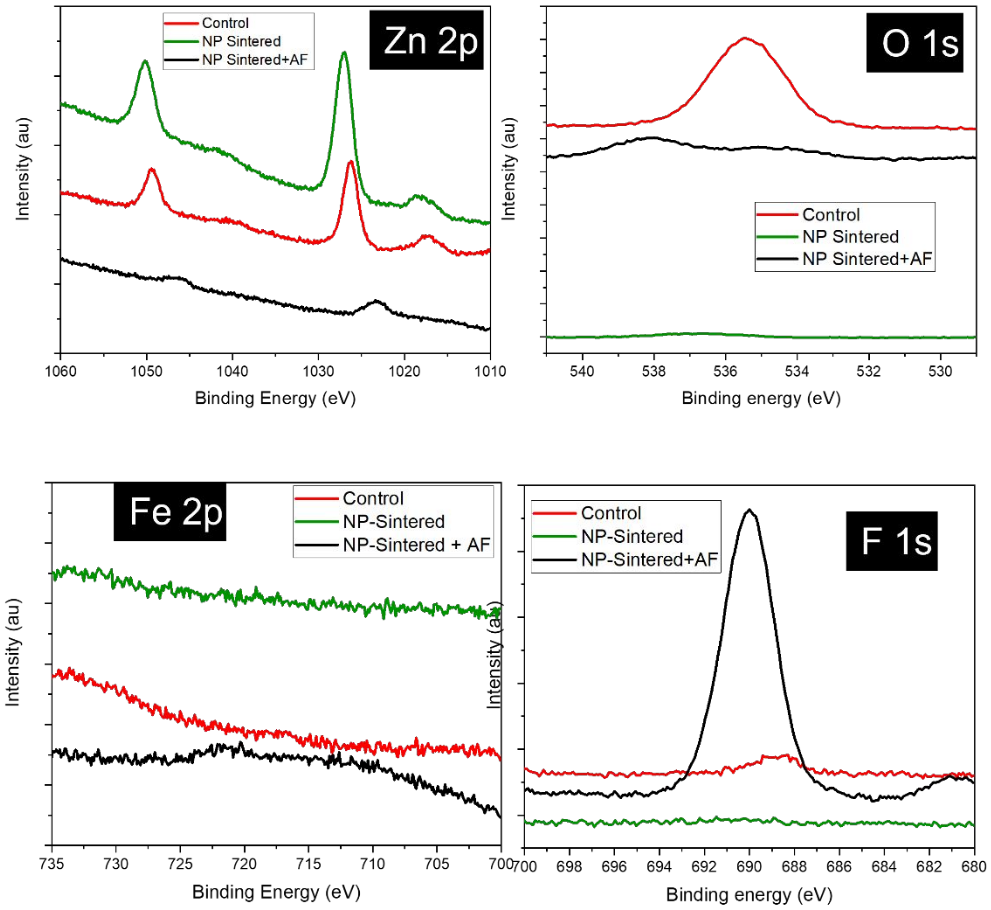

Figure S1 XPS spectra at different binding energy on three representative samples: untreated sample (control) a sintered sample fabricated with a 1g/L solution of ZN-NP and c) the same sintered sample coated with an amorphous fluoropolymer solution, aimed to turn the surface into superhydrophobic. The analysis is focused in the detection of four elements through the signal peaks detected at certain Binding energies: Zn-2p, O-1s, Fe-2p and F-1s.

The samples with higher concentration of Zn on their surface were those sintered with Zn NP. This is expected and it agrees with the EDX results. The sample with the higher concentration of oxygen was the control sample. This can be justified by the presence of ZnO on the sample and agrees well with prior studies [*S. Feliu Jr. *, V. Barranco, Acta Materialia 51 (2003) 5413–5424*]. Note that the zinc nanopowder used for the sintering process is composed by pure zinc (no ZnO) and this is the reason why the O-1s peak is no longer observed after the sintering process. The higher presence of this element once the sample is coated with the fluoropolymer is due to the presence of oxygen within this organic coating. The XPS analysis did not evidence any presence of Fe on any of the samples, since no peaks were observed. It indicates that the surface treatments preserve the Zn coating used to cover the inner steel. These results also agree with our EDX results. Finally, F-1s is only present on the sintered and AF-coated sample, which is also expected due to the high presence of this element on the hydrophobic coating.

2. Durability tests

The durability test used to analyze the physical and chemical resistance of the superhydrophobic samples consists of tape-peeling tests, water immersion tests and UV-ozone exposure. As for the previous section we chose the samples that were textured only by Zn-NP sintering using the solution of 1g/L of nanoparticles in acetone to fabricate them. In all cases, we analyzed how each wearing test affect the wettability properties. Among all the techniques used in our study for characterizing the wettability of the samples we selected the Bouncing Drops Experiments because, as indicated in previous studies [*F. J. Montes Ruiz-Cabello et al. J Colloid Interface Sci 2017, 508.*], drops only bounce on superhydrophobic surfaces for this kind of experiments.

Tape Peeling tests

An adhesive Scotch Tape AM 810t supplied by 3M (adhesion strength of 250N/m) was peeled off the samples using an inclination angle of 90° an fixed velocity of 5mm/s. We proceeded with up to 15 peel-off cycles and monitored the number of bounces measured by bouncing drops experiments. This parameter may be used as a good estimation of the superhydrophobicity degree of the samples. In Figure S2 the results of the percentage of bounces with respect to the initial values (measured for an as-prepared sample) is shown in terms of the number of cycles. Although the loss of wettability response is noticeable, the surfaces maintain their repellency properties since the number of bounces is still higher than 0. Above 15 cycles, we found that the samples maintained their superhydrophobicity.

Since the surfaces are fabricated in a two-step process, we wanted to verify whether the peeling tape tests damage the nanostructures typography, or the hydrophobic coating instead. To address this issue, after 15 peel-off cycles we eliminated the hydrophobic coating by plasma cleaning. Then, we performed another 10 cycles to further test the nanoparticles adhesion to the substrate. In this case, we noticed how the adhesion of the tape to the sample increased, due to the absence of the hydrophobic coating. Finally, we deposited a new layer of the fluoropolymer coating. The results are also shown in Figure S2. We may observe that the samples recover or even improve their wettability performance once they are recoated, which means that the peeling tests do not alter the surface structure. In contrast, the accumulative peel-off cycles damage the hydrophobic layer that is deposit on the samples to low down their surface energy.

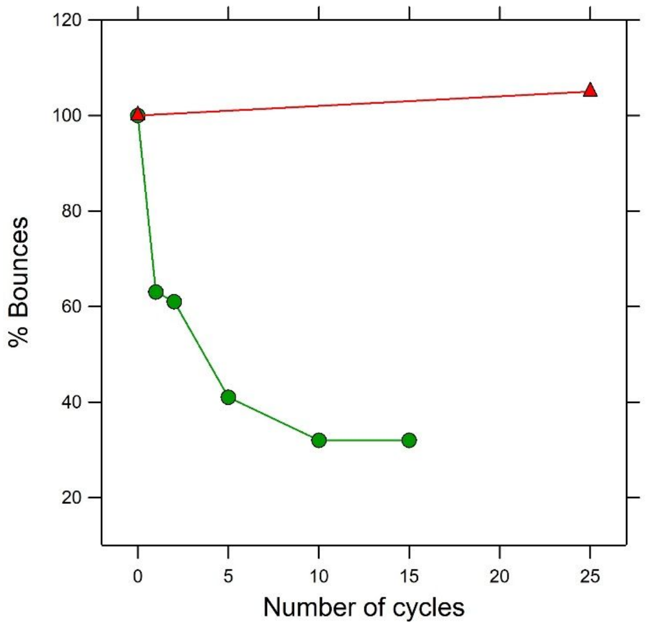

Figure S2 Percentage of Number of Bounces with respect to the original one (measured for fresh samples) given by a drop using Bouncing Drop Experiments. Results are shown in terms of the number of peeling-off cycles. Green circles correspond to samples that were not re-teflonated, while red triangles correspond to samples that were re-teflonated after conducting the wearing test.

UV Exposure tests

The UV resistance of the samples was analyzed using a Novascan UV-Ozone cleaner. The samples were UV-treated for different exposure times up to 60 min. For this wearing test, we again focused on the wettability properties after each sequence, monitored by bouncing drop experiments.

These tests, which results are shown in Figure S3, confirm that the surfaces maintain their repellency properties reasonably well regardless of the exposure time.

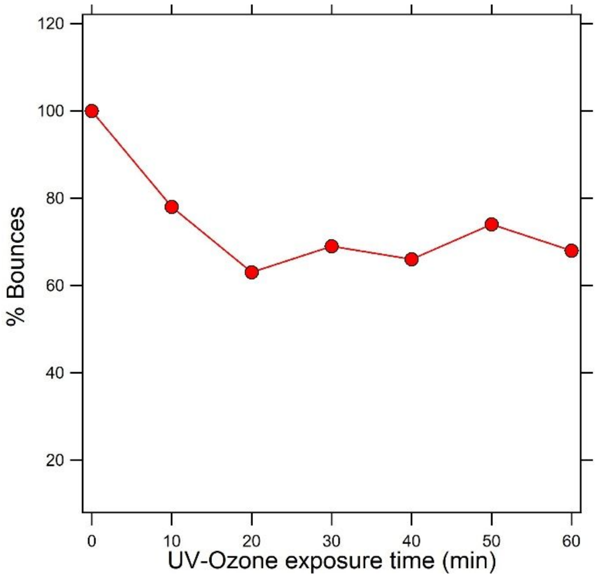

Figure S3 Percentage of Number of Bounces with respect to the original one (measured for fresh samples) given by a drop using Bouncing Drop Experiments. Results are shown in terms the UV-ozone exposure time.

Water immersion tests

A glass crystallizer of 100 mm in diameter was filled with distillate water up to 100 mL approximately. The fresh samples were immersed inside the contained for 24h and we explored how the samples are affected by long water exposure in terms of wetting properties. Once the samples were removed from the container, they were dried with filtered air and then introduced inside an oven for 10 min to eliminate traces of water. We analyzed the number of bounces after these tests and concluded that the samples maintain the 60% in number of bounces with respect to the original sample.

These tests also served to determine whether the sample maintain the original anti-corrosive properties of galvanized steel. In Figure S4 we show how the samples look before the water immersion tests and after 24h of water immersion. We conclude that they both look the same which is evidence of no corrosion or oxidation.

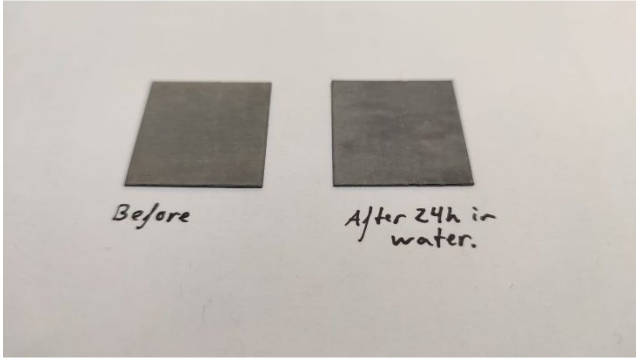

Figure S4 Galvanized steel superhydrophobic samples before and after long water exposure
